# Fibula-Assisted Segment Transport (FAST) for Defect Reconstruction after Resection of Tibial Adamantinoma: Report of Two Treatments

**DOI:** 10.1155/2021/5563931

**Published:** 2021-05-01

**Authors:** A. Rachbauer, A. Laufer, G. Gosheger, G. Toporowski, A. Frommer, E. Jacob, N. Deventer, R. Roedl, B. Vogt

**Affiliations:** ^1^Department of Children's Orthopedics, Deformity Reconstruction and Foot Surgery, Muenster University Hospital, Muenster, Germany; ^2^Department of General Orthopedics and Tumor Orthopedics, Muenster University Hospital, Muenster, Germany

## Abstract

Intramedullary limb lengthening via lengthening nails has been performed for more than three decades to overcome leg length inequalities. Plate-assisted bone segment transport (PABST) has recently been described for the reconstruction of segmental bone defects. We modified this procedure by using the ipsilateral fibula as a “biological plate” and report on its technical particularities and application in the reconstructive treatment of adamantinomas of the tibia in two patients. Both patients were successfully treated by wide resection and reconstruction of the tibial bone via bone segment transport through an expandable intramedullary nail using the remaining ipsilateral fibula to provide stabilization and guidance. This procedure was titled “fibula-assisted segment transport” (FAST). This is a new and promising technique that allows an entirely biological reconstruction of large bone defects of the tibia.

## 1. Introduction

Adamantinoma is a rare low-grade malignant bone tumor with a predilection for the diaphyseal tibia which predominantly affects male patients between the ages of 20 and 50 years [1–3]. In general, there is little soft tissue or fibular involvement, so the fibula can be preserved in most cases. The tumor is primarily diagnosed by radiography, magnetic resonance imaging, and most importantly biopsy, to exclude differential diagnoses such as osteofibrous dysplasia, Langerhans histiocytosis, hemangioendothelioma, fibromas, and bone cysts [2]. It is treated either by amputation or by limb-preserving surgery with wide en bloc resection and surgical reconstruction. The use of a free vascularized fibular transplant and/or structural allograft for reconstruction is considered the most commonly applied treatment modality [4]. Neoadjuvant therapy does not have a place in the treatment of adamantinoma.

Over the past decades, distraction osteogenesis through external fixation has been established as the preferred surgical regimen in the treatment of bone defects [5, 6]. However, due to common complications related to external fixation such as pin tract infection, tedious duration of treatment, soft tissue tethering, and psychological stress for the patient, there are constant attempts to optimize this technique. The external fixator has been combined with a plate or an intramedullary nail for better stabilization [7–9]. Lately, the use of motorized intramedullary nails has become popular, minimizing the adverse effects of external fixation mentioned above [10]. Kahler Olesen has used intramedullary nails for bone segment transport adding a locking plate for stabilization during the distraction process and named this procedure “plate-assisted bone segment transport” (PABST) [11]. Its use has not yet been described in the reconstruction of defects caused by resection of adamantinomas of the tibia.

We report on two patients with adamantinoma of the diaphyseal tibia treated by wide resection of the tumor and reconstruction via bone segment transport without a plate, using the ipsilateral fibula as a “biological plate” instead. This procedure has been titled “fibula-assisted (bone) segment transport” (FAST).

## 2. Case Presentation

### 2.1. Patient 1

**Figure 1:**
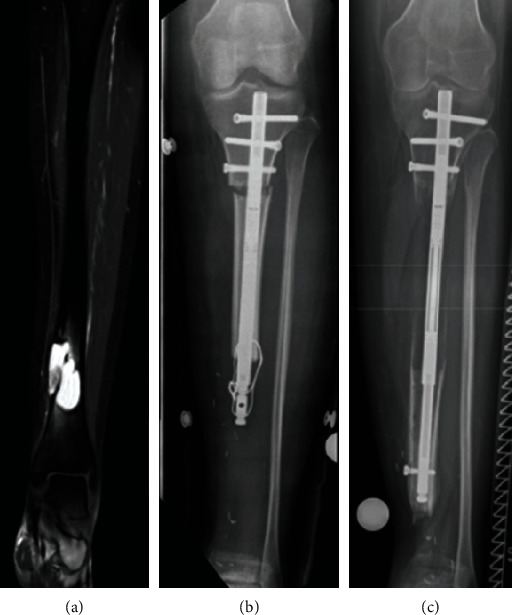
Patient 1. (a) Magnetic resonance imaging showing adamantinoma of the distal tibia. (b) Radiograph showing wide tumor resection preserving the fibula, proximal tibial corticotomy, and implantation of a lengthening nail for bone segment transport. (c) Radiograph showing advanced bone segment transport after exchange to another lengthening nail.

**Figure 2:**
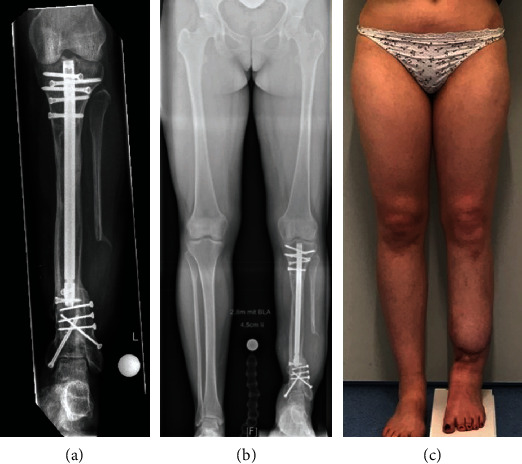
Patient 1. (a) Radiograph showing complete consolidation of the entire tibia including the former callotasis site and the distal docking site with autologous bone grafting using a part of the distal fibular diaphysis as well as trauma nail and multiple screw osteosynthesis. (b) Bilateral long-standing radiograph showing correct alignment but persistent shortening of the reconstructed leg. (c) Clinical presentation showing persistent leg length discrepancy.

A 20-year-old female patient presented with adamantinoma of the distal third of the left tibia. To achieve a wide resection of the tumor, 150 mm of the distal third of the tibia had to be excised, leaving only 20 mm of the intact tibia above the ankle joint. The fibula could be spared. A fibular transposition was considered but deemed to be at high risk for failure due to the small remnant of the distal tibia remaining for stabilization. Therefore, primary amputation or bone segment transport with either a circular external fixator or an intramedullary lengthening nail was proposed. The patient consented to bone segment transport. We performed wide tumor resection followed by antegrade implantation of a magnetically driven intramedullary lengthening nail (PRECICE® limb lengthening system (P10.7-80C245), NuVasive, San Diego, CA, USA) with a maximum stroke of 80 mm. Corticotomy was performed at the proximal tibia. A wire cerclage was applied to fixate the intramedullary nail distally, as screw fixation was not possible due to the short length of the remaining bone segment (Figure 1). Bone transport was started after a latency period of ten days, with a distraction rate of 0.66 mm per day. Progress of treatment was controlled by clinical and radiographic examination at two-week intervals. During distraction, the leg was immobilized in a long-leg cylinder cast. Full weight-bearing was prohibited, mainly because of the instability of the leg but also due to the mechanical features of the distraction nail, which is not load-stable. After achieving a distraction length of 75 mm, the nail was exchanged for another PRECICE® device with the same maximum stroke (P10.7-80C245). As the patient showed insufficient callus formation, the distraction rate had to be reduced to 0.33 mm per day. Unfortunately, this led to premature termination of distraction on behalf of the patient, who was dissatisfied with the prolonged treatment time. Bone transport was thus stopped after having gained an additional distraction length of 50 mm (Figure 2). A docking procedure was performed which required acute shortening of 25 mm through segmental resection of the distal fibular diaphysis to allow docking of the transport segment to the distal tibia. This resulted in a residual leg length discrepancy (LLD) of 50 mm. Stabilization of the tibia was achieved through an intramedullary trauma nail (TRIGEN™ META-NAIL™ Tibial Nail System, Smith+Nephew, Watford, UK). The resected part of the fibula was used as an autograft at the docking site. For additional stabilization, a distal tibiofibular cross-fusion was established through screw fixation. Postoperatively, weight-bearing was prohibited, and additional casting was applied for another six weeks. After removal of the cast, the patient was provided with a custom-made orthotic brace. The bone regenerate showed delayed consolidation, presumably at least partly caused by the smoking habits of the patient. Due to the delayed consolidation, full weight-bearing was permitted only after twelve weeks. Full consolidation was observed after 75 weeks.

The distraction index (length of distraction achieved in mm divided by the duration of distraction in days) of the bone transport was 0.32 mm/day, and the consolidation index (months from index surgery until consolidation divided by the length of the regenerate in cm) was 2.24 months/cm.

**Figure 3:**
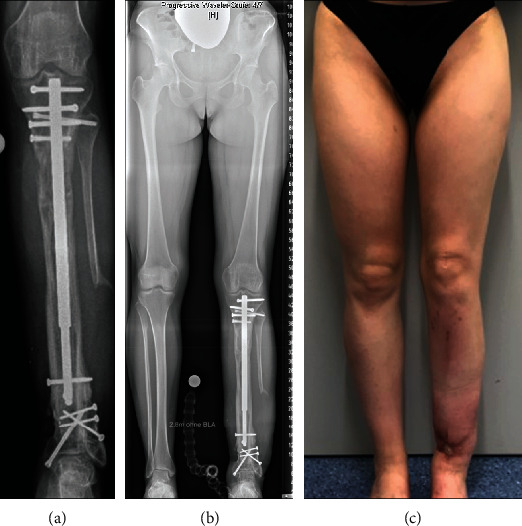
Patient 1. (a) Radiograph showing complete consolidation of the proximal tibial callotasis site after exchange to another lengthening nail for distraction. (b) Bilateral long-standing radiograph showing correct alignment and equalization of leg lengths. (c) Clinical presentation showing equal leg lengths.

Two years after termination of bone transport, the patient desired equalization of the residual leg length discrepancy of 50 mm. The trauma nail was therefore exchanged for another PRECICE® lengthening device (PRECICE STRYDE® (PS10.0-80SJ265)). This implant allowed full weight-bearing. Leg length equality was achieved after distraction of 50 mm. Full consolidation of the bone regenerate was observed after 17 weeks (Figure 3). The distraction index was 0.56 mm/day, and the consolidation index was 1.40 months/cm.

At the time of the last follow-up, the patient was overall satisfied with the functional result, presenting a free range of motion of the knee and ankle joints with an extension/flexion of 0/0/110° of the knee joint and an extension/flexion of 5/0/20° of the ankle joint. The patient was able to bear full weight without pain. There was no indication of a torsional deformity (symmetrical foot-thigh angle of 25°). The mechanical axis deviation (MAD) was -7 mm, the medial proximal tibia angle (MPTA) was 90°, and the lateral distal tibial angle (LDTA) was 90°. To date, a total of six surgeries has been performed ((1) biopsy, (2) wide tumor resection and nail implantation, (3) exchange of the lengthening nail, (4) exchange for a trauma nail, (5) exchange for a lengthening nail, and (6) removal of all implants including the lengthening nail). 4.5 years after the first diagnosis, the patient is disease-free, with no signs of tumor dissemination or local tumor recurrence, presenting fully restored function of the leg.

### 2.2. Patient 2

**Figure 4:**
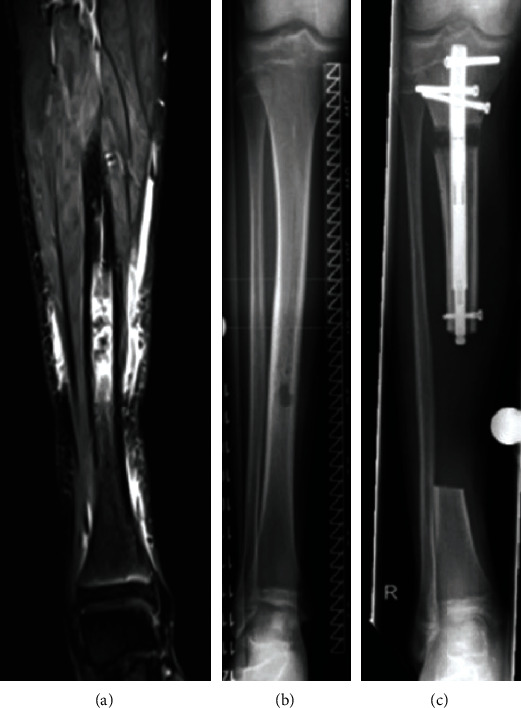
Patient 2. (a) Magnetic resonance imaging showing adamantinoma of the diaphyseal tibia. (b) Radiograph showing adamantinoma of the diaphyseal tibia. (c) Radiograph showing wide tumor resection preserving the fibula, proximal tibial corticotomy, and implantation of a lengthening nail for bone segment transport.

**Figure 5:**
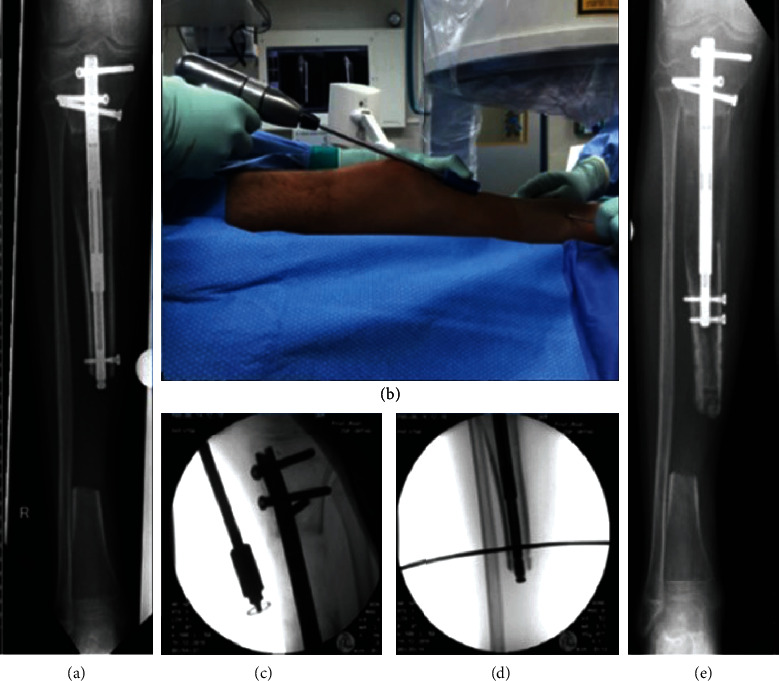
Patient 2. (a) Radiograph showing proceeded bone segment transport. (b–d) Clinical and fluoroscopic presentation of intraoperative retraction of the nail using the fast distractor tool with temporary K-wire transfixation of the transport segment. (e) Radiograph showing advanced bone segment transport.

A 14-year-old male patient presented with adamantinoma of the right diaphyseal tibia (Figure 4). Fibular transfer or bone segment transport by either an external fixator or a lengthening nail was proposed to the patient and his family, who consented to bone transport. After 120 mm of tibial resection, a PRECICE® intramedullary lengthening nail with a maximum stroke of 50 mm (P10.7-50J180) was implanted through an antegrade approach; corticotomy was performed at the proximal tibia. The fibula could be preserved. Distraction commenced at the tenth postoperative day with a distraction rate of 0.66 mm per day. After distraction of 40 mm, revision surgery was performed to temporarily release the nail distally and retract the nail using the fast distractor tool (Fast Distractor, NuVasive, San Diego, CA, USA) to allow further distraction (Figure 5). To prevent loss of correction, the transport segment was temporarily transfixed through a tibiofibular K-wire. After having gained another 40 mm of distraction, this procedure was repeated. The full length of the lower leg was reestablished after in total 120 mm of distraction, and the PRECICE® nail was replaced by an intramedullary trauma nail (TRIGEN™ META-NAIL™). At the docking site, a supplementary locking plate was implanted (VariAx Foot Locking Plate System, Stryker, Kalamazoo, MI, USA), and autologous cancellous bone grafting harvested from the ipsilateral iliac crest was performed. The distraction index was 0.60 mm/day, and the consolidation index was 0.75 months/cm.

**Figure 6:**
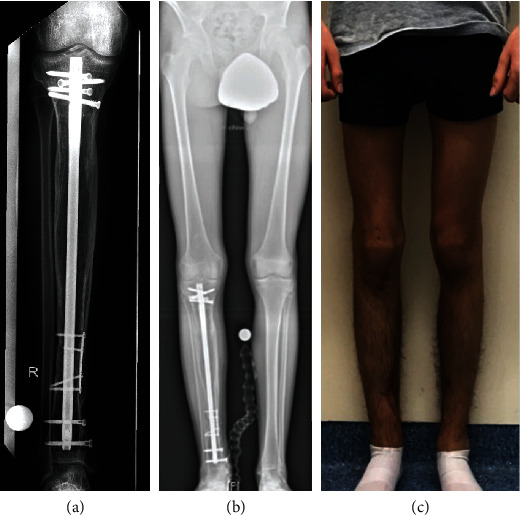
Patient 2. (a) Radiograph showing complete consolidation of the entire tibia including the former callotasis site and the distal docking site with autologous bone grafting harvested from the iliac crest as well as trauma nail and locking plate osteosynthesis. (b) Bilateral long-standing radiograph showing correct alignment and length of the reconstructed leg. (c) Clinical presentation showing equal leg lengths.

After eight weeks, the docking site and the callotasis site showed sufficient osseous consolidation; thus, full weight-bearing was permitted (Figure 6). Three months later, the patient returned to sports. At the time of the last follow-up, the patient was overall satisfied, reporting no restrictions in activities of daily living.

The range of motion of the knee joint was unimpaired with an extension/flexion of 0/0/120°, and the ankle joint presented an extension/flexion of 15/0/20°. There were no signs of a torsional deformity with a symmetrical thigh-foot angle of 20°. The MAD was +6 mm, the MPTA was 88°, and the LDTA was 90°.

In total, five surgeries were performed ((1) biopsy, (2) wide tumor resection and implantation of the lengthening nail, (3 and 4) reloading of the nail for further distraction, and (5) docking surgery and nail exchange). Two years after the initial diagnosis, the patient is still disease-free with no signs of local tumor recurrence or dissemination and presents excellent function of the operated leg. Removal of the implants is pending.

## 3. Discussion

Distraction osteogenesis through external fixators has been established in the treatment of bone defects over the past 50 years [5, 12]. Among other advantages, this method allows full weight-bearing during treatment, has minimal risk of epiphyseal damage in skeletally immature patients, and is applicable even in short bone segments or those close to the joint. Moreover, residual LLD after segment transport can be addressed by distraction osteogenesis at any time. However, the procedure is associated with adverse effects such as pin tract infections, muscle and joint contractures, joint dislocation, vascular injuries, hypertrophic scarring, tedious duration of treatment, risk of fractures after frame removal, and patient non-compliance, not least because of psychological stress caused by the treatment itself [6, 13].

There has been constant development in recent years aiming at shortening treatment duration, e.g. through integrated fixation by combining the external fixator with an intramedullary nail or a plate [7–9]. It was reported that the time in the frame could be reduced by approximately 30%, since the removal of the frame is possible before full consolidation is achieved due to the internal stabilization provided through the plate and nail, respectively. The occurrence of secondary deformities is reduced as well, but reportedly, there is a higher risk of deep infections, regardless of the technique applied (external fixator combined with either a plate or nail) [7–9, 14]. Quinnan and Lawrie described the use of a cable wire in combination with external fixation for cable bone transport. They reported a low number of true complications but observed a significant number of obstacles which led to unplanned revision surgeries [15].

Fully implantable expandable intramedullary nails have recently become increasingly popular, as they minimize adverse effects attributed to external fixators such as soft tissue tethering, pain, and pin tract infection [10]. In 1997, Baumgart et al. reported on a case of a 26-year-old patient with Ewing's sarcoma of the tibia in whom bone transport was performed through a motorized intramedullary nail (Fitbone®, Wittenstein, Igersheim, Germany) [16]. A similar case was reported by Kold and Christensen in 2014, even though in this case the segmental bone defect was of traumatic origin [17]. The Fitbone® bone transport nail is custom-made, so a certain production time has to be taken into account when planning treatment. Furthermore, weight-bearing is not possible during the distraction and consolidation period, as opposed to treatment with an external fixator [18].

Another bone transport technique was introduced by Krettek in 2018. The author described the combination of a so-called MagicTube (K-Implant GmbH, Garbsen, Germany) and a motorized lengthening nail. After bone transport has been performed through the lengthening nail, further distraction can be achieved through the MagicTube [19]. However, this device is not commercially available yet.

In 2011, NuVasive released a magnetically driven intramedullary lengthening nail which is available in standardized diameters (PRECICE® limb lengthening system) [20]. To date, there are few reports on its use for bone segment transport. Among others, Kahler Olesen has used the PRECICE® nail for bone segment transport, applying an additional locking plate for stabilization during the distraction period (PABST) [11]. Wright et al. in 2019 reported on a new bone transport procedure to address the posttraumatic segmental bone loss of the femur. Similar to the PABST procedure, they combined a lengthening nail with a locking plate but used two instead of one plate in order to stabilize the femur and allow full weight-bearing and to prevent the development of secondary coronal deformities [21]. Just recently, Zeckey et al. described the application of the new PRECICE® Bone Transport System, through which bone transport can be performed without requiring additional stabilization, e.g. through a plate [22]. The preliminary results seem to be promising, but the long-term outcomes in particular regarding complication rates and functional results are still due. Since the PRECICE® Bone Transport System has been released only recently, it was not yet available for the treatment of the two patients presented in this case series.

In reconstructive treatment following wide tumor resection of the diaphyseal tibia, as is the case in the treatment of adamantinomas, restoration of bone integrity can be achieved with a vascularized fibula transfer and/or structural allograft [4]. Endoprosthetic bone replacement, on the other hand, has been described in few cases, but the outcome was mostly found to be unsatisfactory [4, 23, 24]. In comparison to endoprosthetic replacement, biological reconstruction offers the advantage of reduced risk of infection and superior functional outcome [25, 26]. However, reconstructive treatment often requires several additional surgeries; thus, the treatment itself is tedious. Nevertheless, in our experience, the requirement of revision surgeries after successful termination of reconstruction is unlikely.

To our knowledge, bone transport via an expandable nail in the treatment of segmental bone loss following resection of adamantinoma of the tibia has not yet been described in the literature, even though this procedure has been reportedly applied in the treatment of other (malignant) primary bone tumors [16, 27].

Contrary to the PABST procedure, we refrained from applying an additional plate for stabilization, because the preserved and intact fibula served as a “biological plate.” In this way, the fibula not only provided guidance during distraction but also indicated the required length of the bone transport in order to achieve leg length equalization. In contrast to reconstructive approaches using the free vascularized fibula, the FAST procedure eliminates the risk of donor site morbidity and nonunion of the tibia and the fibular graft.

Successful implementation of distraction osteogenesis prior or concomitantly to chemotherapy has been described in several case reports [28–30]. However, as chemotherapy and radiation are reportedly associated with adverse effects on bone formation and consolidation, distraction osteogenesis in the management of bone tumors that require further adjuvant treatment should be considered carefully [31–34]. In addition, the psychological aspect of the tedious process of bone transport requiring high patient compliance and regular controls at the hospital have to be taken into account. Nevertheless, even though treatment duration was lengthy, eventually, sufficient consolidation of the bone regenerate was achieved in both our patients. Furthermore, we observed a similar consolidation index in comparison to the results reported for external fixator controlled segment transport and for the PABST procedure [12, 27, 35].

Due to primary instability, full weight-bearing is not possible in most internal techniques. In addition, application of a cast or orthotic brace is required in FAST to gain further stabilization, which is not necessary for the PABST procedure. Nevertheless, the additional locking plate required in PABST causes more trauma to the soft tissue and thus could increase the risk for device-associated infection and a prolonged healing period.

Compared to bone segment transport through external fixation, in FAST, most additional surgeries which are required subsequent to bone transport (such as exchange nailing) can be anticipated and planned in advance, depending on the transport distance. Therefore, thorough informed consent with the patient should be obtained prior to treatment initiation to ensure good patient compliance.

In case of segmental bone defects of the tibia following wide tumor resection with the preserved fibula, bone transport via an expandable intramedullary nail using the ipsilateral fibula for stabilization and guidance is a viable option to achieve restoration of bone integrity and reconstitution of the original leg length. The FAST technique allows a complete biological reconstruction and thus eliminates the risks implied by implantation of endoprostheses or diaphyseal implants. Common complications associated with bone transport through external fixation and free fibular transfer can be obviated. However, the requirement for multiple (minor) revision surgeries should be taken into account, and the patient needs to be informed elaborately about the procedure and its potential pitfalls.

## Data Availability

The datasets used and/or analyzed during the current study are available from the corresponding authors on reasonable request.

## Additional Points

*Reporting Guidelines*. The authors followed the CARE guidelines, and the CARE checklist is provided as an additional file.
